# The effect of n-3 polyunsaturated fatty acids on incidence and severity of oxaliplatin induced peripheral neuropathy: a randomized controlled trial

**DOI:** 10.1186/s40364-016-0066-3

**Published:** 2016-06-23

**Authors:** Ali Esfahani, Mohammad hossein Somi, Hormoz Ayromlou, Alireza Nikanfar, Mohammad Asghari Jafarabadi, Bina Eftekhar Sadat, Zohreh Ghoreishi

**Affiliations:** Gastroenterology, Liver & Gastrointestinal Diseases Research Center, Tabriz University of Medical Sciences, Tabriz, Iran; Neurosciences Research Center, Tabriz University of Medical Sciences, Tabriz, Iran; Hematology and Oncology Research Center, Tabriz University of Medical Sciences, Tabriz, Iran; Tabriz Health Services Management Research Center and Department of Statistics and Epidemiology, Faculty of Health and Nutrition, Tabriz University of Medical sciences, Tabriz, Iran; Physical Medicine & Rehabilitation Research Center, Tabriz University of Medical Sciences, Tabriz, Iran; Nutrition Research Center, Tabriz University of Medical Sciences, Tabriz, Iran

**Keywords:** Colon cancer, n-3 polyunsaturated fatty acids, Oxaliplatin, Neuropathy

## Abstract

**Background:**

Oxaliplatin induced peripheral neurotoxicity (OXIPN) is the major dose-limiting and long-lasting side effect of oxaliplatin. N-3 PUFAs have neuroprotective property via their effects on voltage-gated ion channels and by reducing the production of proinflammatory cytokines that causes neuropathy. This study was a randomized double blind placebo controlled trial to find the possible advantages of n-3 PUFAs for preventing and reducing the severity of OXIPN in patients with colon cancer.

**Methods:**

Eligible patients with colon cancer randomly allocated to take n-3 PUFAs pearls, 640 mg t.i.d during chemotherapy with oxaliplatin and one month after the cessation of the treatment or placebo. All patients were evaluated for incidence and severity of OXIPN based on “reduced Total Neuropathy Score” in which clinical and electrophysiological assessments were included.

**Results:**

Seventeen patients (47 %) of the n-3 PUFA supplemented group (*n* = 36) did not develop PN while it was 11 %(4 patients) in the placebo group (*n* = 35). There was a significant difference in PN incidence (OR = 0.14, .95 % CI = (0.04 to 0.49), *p* = 0.002). The difference of OXIPN severity was significant between the two study groups (B = −1.61, 0.95 % CI = (−2.59 to −0.62), *p* = 0.001).

**Conclusions:**

N-3 PUFAs may have neuroprotective effect for reducing the incidence and severity of OXIPN. Finding an effective prophylactic or symptomatic therapy for OXIPN would significantly improve the patients’ quality of life.

**Trial registration:**

IRCT201112158397N2

## Background

Colorectal cancer (CRC) is one of the most common cancers in the world with a considerable mortality rate and rising incidence during the last decades [1]. Oxaliplatin is now a central choice for treatment of advanced CRC [2] as the first line chemotherapy regimen [3] and as adjuvant therapy [4]. However, it induces a peripheral neuropathy which is the major dose-limiting side effect of this chemotherapeutic agent. Oxaliplatin-induced peripheral neuropathy (OXIPN) results in an acute transient syndrome, and a chronic sensory-motor axonal peripheral neuropathy. It may be a dose-limiting and disabling neurotoxicity that continues for a long time [5–8].

Eicosapentaenoic acid (EPA) and docosahexaenoic acid (DHA) are two main forms of n-3 polyunsaturated fatty acids (PUFAS). They are structural components of cell phospholipid membranes including those of the nervous system [9]. They have beneficial effects on a number of psychiatric and neurodegenerative diseases [9–11]. At present, there is no neuroprotective agent for prophylaxis and treatment of OXIPN [5, 12, 13], although several compounds have been proposed to have some effects on acute form of OXIPN. We previously reported the efficacy of n-3 PUFAs for prophylaxis against paclitaxel-induced peripheral neuropathy in patients with breast cancer [14].

The possible advantages of n-3 PUFAs for preventing and reducing the severity of OXIPN in patients with colon cancer were assessed in the present study that is the first attempt in this area. As CRC survivors are the third largest population of cancer survivors [2, 5, 13], attenuating the risk of OXIPN- the major factor that decreases their quality of life- is now a major concern. Moreover, the serum level of proinflammatory cytokines was measured for assessing their changes during supplementation with n-3 PUFAs.

## Methods

### Primary outcome

This randomized double-blind placebo-controlled trial was conducted to assess the effect of n-3 PUFAs on incidence and severity of OXIPN in patients with colon cancer undergoing chemotherapy with oxaliplatin.

### Patients

From February 2012 to January 2015, male or female patients with confirmed stage III colon cancer, aged between 20 and 85 years, and treated at Sheikhorrais University Clinic with oxaliplatin, 130 mg/m^2^ IV, administered over 2 h infusion in day 1 and oral capecitabine (1000 mg/m^2^, b.i.d) on days 1–4, to be repeated every 3 weeks for 8 cycles, were enrolled. Other inclusion criteria of the study were WHO performance status score of 0-1, and normal kidney and liver function.

Patients were excluded from the study if they had pre-existing peripheral neuropathy (PN) due to diabetes mellitus, alcoholic disease, HIV, and inherited PN- associated disorders. Patients who received other neurotoxic drugs prior to oxaliplatin therapy or those who were on any form of supplement therapy (fish oil, vitamins and minerals) were also excluded from the study.

### Intervention and randomization

Eligible patients were randomly allocated in a 1:1 ratio to receive n-3 PUFAs pearls (Mor DHA Mini I.Q. Minami Nutrition NV, Drie Eikenstraat 661, 2650 Edegem, Belgium) at a dose of 640 mg (54 % DHA, 10 % EPA) three times a day coinciding with the start of chemotherapy until one month after the end of treatment or placebo pearls containing sun flower oil (Dana Pharma, Tabriz, Iran). Placebo and n-3 PUFAs pearls were similar in appearance and color and administered based on the same protocol.

Random allocation software (RAS) version 1.0.0. (Esfahan, Iran) was used for Permuted block randomization to assign patients to either the n-3 PUFAs supplemented group or the control group. Only the randomization coordinator of the study was aware of the patients’ allocations.

### Neurologic evaluation

All patients were evaluated prior to chemotherapy and one month after the cessation of treatment (about 25 weeks) by the same neurologist who carried out both clinical and electrophysiological assessments. He was blinded to the patient’s allocation to study groups.

The presence of chronic OXIPN and grading of its severity was ascertained by reduced Total Neuropathy Score (TNSr) as the primary outcome measures. TNSr is an easy to use composite scale for assessing chemotherapy-induced peripheral neuropathy (CIPN) [15]. Subjective sensory symptoms, pin sensibility, deep tendon reflexes, and sural and peroneal amplitude (a total of 7 parameters) are all included in TNSr. Each parameter has a score from 0 to 4, based on the measured severity; then the total score ranges from 0 to 28 that grades OXIPN as follows: grade 0, no OXIPN, 1–10, mild; 11–19, moderate; and 20–28, severe OXIPN [14].

### Secondary outcome

- Nerve conduction studies (NCVs)
  Electrophysiological studies were carried out unilaterally (right side) under a uniform protocol using a Nicolet/VIASYS Viking Quest EMG Machine [16]. Thirty-two to 34^°^C was set as the distal skin temperature. The estimated parameters included: Distal motor latency (DML), peak to baseline amplitude of compound muscle action potential (a-CMAP), and motor conduction velocity for tibial, Peroneal and ulnar nerves. Moreover, peak-to-peak amplitude of sensory action potentials (a-SAP) and sensory conduction velocity (antidromic technique) of sural and ulnar nerves were also measured.
- Proinflammatory cytokines
  Serum levels of proinflammatory cytokines: interleukin-6 (IL-6), tumor necrosis factor alpha (TNF-α), and high-sensitivity C-reactive Protein (hs-CRP) were measured before the onset of chemotherapy and one month after the cessation of treatment by Human IL-6 Platinum ELISA, Human TNF alpha Platinum ELISA (eBioscience, INC. San Diego, CA 92121, USA) and Minineph™ Human kits (Birmingham, UK) respevtively.

### Protocol approvals and patient consent

This study (IRCT201112158397N2) was approved by the Ethics Committee of Tabriz University of Medical Sciences (No: 5/4/10130) and informed consent was obtained from all the study subjects before any intervention.

### Statistical analysis

Data have been shown using mean (SD) and frequency (percent) for quantitative and qualitative variables respectively. To define the differences of nerve conduction measurements, analysis of covariance was used adjusting baseline measurements. Logistic regression analysis was performed to find the difference of OXIPN incidence between the two groups, estimating the odds ratio. Ordinal regression analysis was done to compare the severity of OXIPN. All tests were two-tailed and significance level was determined 0.05. For analyzing the results on an intention to treat (ITT) basis, multiple imputation was performed by related variables. Statistical power of the study was 80 % and all analysis was done using spss software, (spss Inc., Chicago, IL).

### Sample size

To determine the sample size, primary information was obtained from the study by Ghoreishi et al. In this study, the protective effect of n-3 PUFAs against paclitaxel-induced neuropathy in patients with breast cancer was assessed. Using odds ratio (OR) = 0.3, α = 0.05, a power of 80 %, and two-tailed analysis and G Power software (Franz Foal, Kiel University, Germany), the minimum sample size was estimated 38 subjects and after considering 30 % lost to follow up, it was increased to 50.

## Results

### Patients

**Fig. 1:**
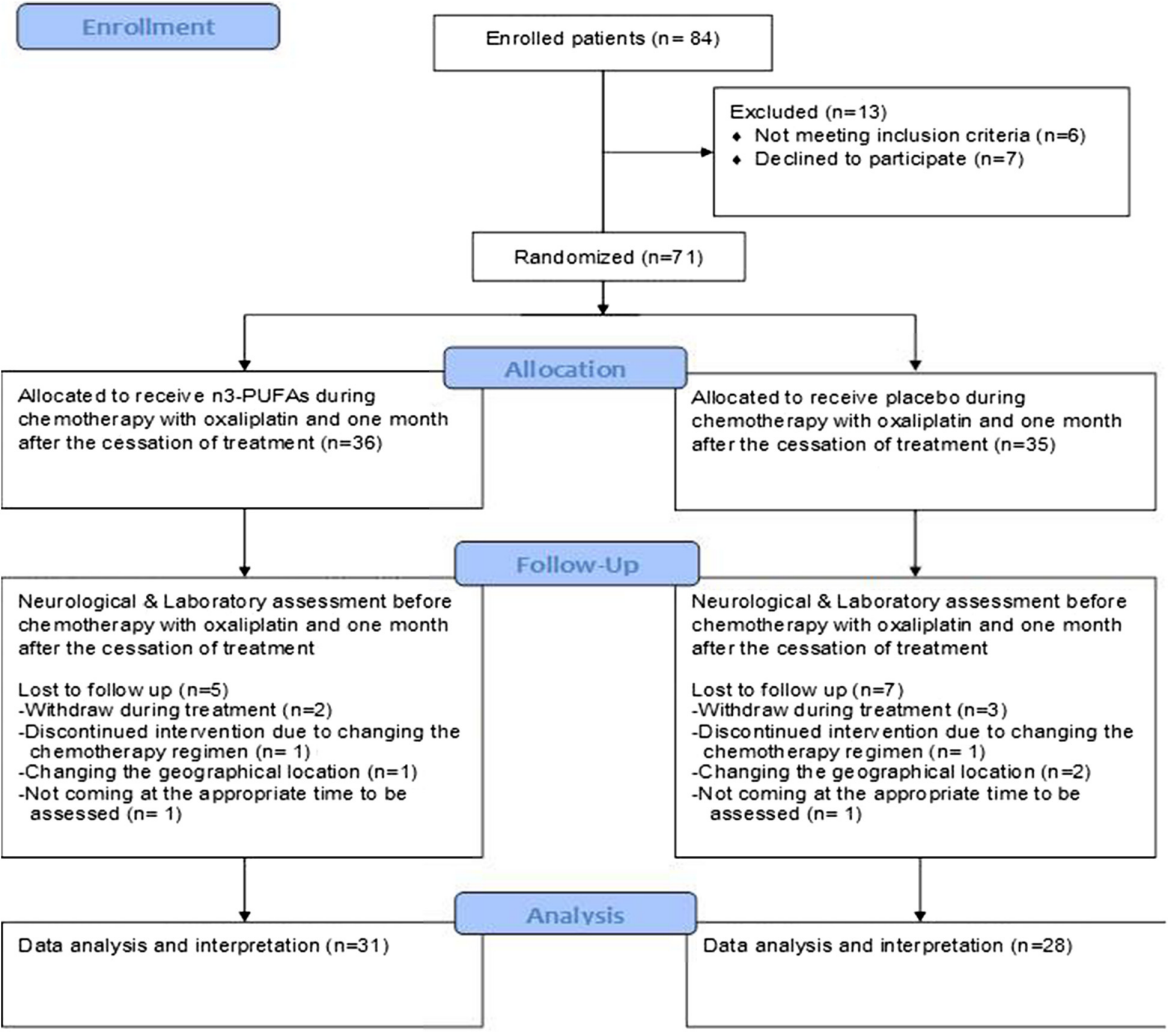
Flowdiagram of the study

Among the 84 recruited patients, 13 patients were excluded from the study due to diabetes mellitus, declined to participate, and taking some kinds of oral supplements (Fig. 1). Therefore, 71 patient were randomly allocated to receive n-3 PUFAs (*n* = 36, 50 % male with the average of age and BMI as: 54.14 ± 10.53 & 23.47 ± 3.40 kg/m^2^ respectively) or placebo (*n* = 35, 60 % male, with the average of age and BMI as: 53.40 ± 15.70 & 23.22 ± 4.77 kg/m^2^ respectively). There was not any statistical difference between two groups in terms of gender, age, and BMI (*P* = .477, .816, and .806 respectively, Table 1). The rate of drop out was 5 patients in n-3 PUFAs supplemented group and 7 patients in the placebo group (Fig. 1), so the response rate was 80.09 %. Data of the main outcome (incidence and severity of OXIPN), was divided into 2 categories: code 1 allocated to missing and code 2 allocated to valid observations. Then demographic variables were compared between ITT patients and efficacy population. Accordingly, they were not statistically different in terms of age (*P* = 0.658), BMI (*P* = 0.224) and gender (*P* = 0.354).

**Table 1:** General characteristics of the patients

	Group		
	n-3 PUFAs (*n* = 36)	Placebo (*n* = 35)	*P* value
Gender (%)			
Male	50	60	
Female	50	40	.477^*^
Age (Year, mean ± SD)	54.14 ± 10.53	53.40 ± 15.70	.816^**^
Male	58.22 ± 8.02	54.48 ± 16.56	
Female	50.06 ± 11.34	51.79 ± 14.78	
BMI^a^ (kg/m^2^,mean ± SD)	23.47 ± 3.40	23.22 ± 4.77	.806^**^
Male	22.43 ± 2.94	22.26 ± 2.91	
Female	24.54 ± 4.68	24.91 ± 6.77	

*P value is reported based on exact Chi-square test

**P value is reported based on Independent-Samples *T* Test

^a^Body mass index

All patients suffered from metastatic colon cancer (stage III) and they treated with oxaliplatin, 130 mg/m^2^ IV, administered over 2 h infusion in day 1 and oral capecitabine (1000 mg/m^2^, b.i.d) on days 1–4, to be repeated every 3 weeks for 8 cycles. No patient received radiation therapy. The patients of the two groups were equivalent regarding the treatment protocol.

### Assessment of OXIPN

**Fig. 2:**
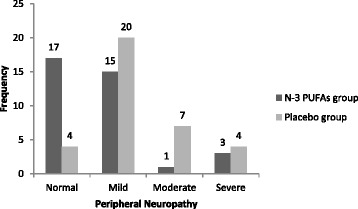
Oxaliplatin induced peripheral neuropathy in the study groups

One month after the cessation of chemotherapy, 17 patients (47 %) did not develop OXIPN in n-3 PUFAs supplemented group while the remaining 19 patients manifested some grade of neurotoxicity based on TNSr: mild OXIPN in 15(42 %) patients; moderate and severe OXIPN in 1(3 %) and 3(8 %) patients respectively. In placebo group, OXIPN was disclosed in 31 (88 %) of the patients: mild OXIPN in 20 patients (57 %), moderate and severe OXIPN in 7 patients (20 %) and 4 patients (12 %) respectively (Fig. 2). A significant difference was observed between the two groups in OXIPN incidence (OR = 0.14, .95 % CI = (0.04 to 0.49), *p* = 0.002). So group I had 86 % lower risk of PN incidence. The number needed to treat (NNT) to prevent one PN event was 2, and absolute risk reduction(ARR) for supplemented group compared with the control group was 0.41(0.16 to 0.59). Remarkably, there were a statistically significant difference in severity of OXIPN between these two groups (B = −1.61, .95 % CI = (−2.59 to −.62), *p* = 0.001). Adjusting for age, the differences between incidence and severity of OXIPN were more significant [(OR = 0.11, .95 % CI = (0.27 to 0.42), *p* = 0.001); (B = −1.74, .95 % CI = (−2.74 to − .75), *p* =0.001)].

### Nerve conduction studies

Significant statistical changes were observed between two study groups in terms of tibial and ulnar motor a-CMAP at one month after the cessation of chemotherapy (Table 2). However, the percent change measured (final measures versus the baseline values) showed that the deterioration of tibial, and ulnar a-CMAP was considerable in placebo group while they were increased in the. Moreover, the increase of peroneal nerve a-CMAP in the n-3 PUFAs group was much more than the increase in the placebo group (Table 2).

**Table 2:** Comparing the motor nerve conduction measurements between the two study groups

	n-3 PUFAs (Mean ± SD)	Placebo (Mean ± SD)	Mean Difference	95 % CI, *P*-value
Tibial nerve				
DML 1 (ms)	4.82 ± .81	5.80 ± 1.31	−.98	−1.50 to −.46, <.001*
DML 2^a^	4.60 ± .79	5.70 ± .95	− .68	− 1.18 to −.17, .009**
% Change^c^	− 4.77	− 1.73		
a-CMAP1(mV)	9.30 (5.62–13.72)	10.20 (5.60–15.30)	--	.696***
a-CMAP 2	10.20 (6.20–13.00)	6.10 (3.47–9.25)		.003^b^
% Change	9.68	− 40.20		
MCV 1(m/s)	45.81 ± 6.61	43.06 ± 5.08	2.74	−.05 to 5.54, .054*
MCV 2	45.67 ± 6.18	42.34 ± 4.68	1.86	− .52 to 4.25, .123**
% Change	−.31	− 1.70		
Peroneal nerve				
DML 1 (ms)	4.34 ± .92	5.21 ± 1.44	−.87	−1.45 to −.30, .004*
DML 2	4.16 ± 1.04	5.02 ± 1.95	.08	− .78 to .94, .850**
% Change	− 3.93	− 3.65		
a-CMAP1(mV)	4.00 (3.00–4.60)	2.80 (1.90–5.20)	--	.238***
a-CMAP 2	4.40 (3.10–5.30)	2.90 (1.17–4.75)		.217^b^
% Change	10.00	3.57		
MCV 1(m/s)	47.59 ± 5.95	44.07 ± 6.91	3.51	.40 to 6.63, .028*
MCV 2	44.70 ± 5.30	43.60 ± 6.87	− 1.37	− 3.88 to 1.14, .278**
% Change	− 6.07	−1.07		
Ulnar nerve				
DML 1 (ms)	3.12 ± .61	3.35 ± .59	−.23	−.51 to .060, .118*
DML 2	2.97 ± .55	3.33 ± .59	− .26	− .54 to .02, .073**
% Change	− 4.81	− .60		
aCMAP1(mV)	11.85 (9.40–14.30)	12.40 (10.80–13.10)	--	.394***
a-CMAP 2	13.10 (10.10–15.00)	10.75 (10.00–13.60)		.033^b^
% Change	10.55	− 13.31		
MCV 1(m/s)	63.01 ± 26.22	55.60 ± 10.89	7.42	−2.14 to 16.98, .126*
MCV 2	53.64 ± 16.72	53.98 ± 6.34	.07	−6.84 to 6.97, .984**
% Change	− 14.87	− 2.90		

*DML* distal motor latency, *a-CMAP* amplitude of compound muscle action potential, *MCV* motor conduction velocity

*P value is reported based on Independent-Samples *T*-Test

**P value is reported based on the analysis of covariance, adjusted for baseline values

***P value is reported based on the Mann–Whitney *U* Test

^a^One month after the cessation of chemotherapy

^b^ *P* value is reported based on the Mann–Whitney *U* Test (for changes of values during the intervention period)

^c^Percent change in proportion to the baseline values

There was not a statistically difference between the two study groups according to sensory conduction studies, although a considerable trend for the differences of sural a-SAPs was observed between the two groups. The percent change of both sural and ulnar nerves’ a-SAP showed a considerable decrease however, the decrease of the a-SAPs was strongly more pronounced in the placebo group at one month after the cessation of chemotherapy (Table 3).

**Table 3:** Comparing the sensory nerve conduction measurements between the two study groups

	n-3 PUFAs (Mean ± SD)	Placebo (Mean ± SD)	Mean Difference	95 % CI *P*-value
Sural nerve				
aSAP1(μV)	9.95 (5.52–16.40)	5.50 (3.40–14.10)	--	.033***
aSAP 2^a^	6.30 (4.60–10.30)	.00 (.00–7.32)		.052^b^
%Change^c^	− 36.68	− 100.00		
SCV1(m/s)	43.99 ± 15.17	44.82 ± 6.27	−.83	−6.44 to 4.78, .766*
SCV 2	42.29 ± 15.51	29.67 ± 13.01	12.24	2.22 to 22.26, .018**
% Change	−3.84	− 33.80		
Ulnar nerve				
aSAP1(μV)	27.90 (16.82–42.77)	17.50 (12.40–35.50)	--	.144***
a- SAP 2	22.80 (6.20–27.30)	10.14 (2.70–18.60)		.430^b^
% Change	− 18.28	− 42.06		
SCV1(m/s)	51.66 ± 8.34	50.15 ± 8.23	1.50	−2.51 to 5.52, .457*
SCV 2	44.08 ± 11.14	42.62 ± 7.26	.98	−4.61 to 6.57, .726**
% Change	− 14.66	− 15.01		

*a-SAP* sensory action potential amplitude, *SCV* sensory conduction velocity

*P value is reported based on Independent-Samples *T*-Test

**P value is reported based on the analysis of covariance, adjusted for baseline values

***P value is reported based on the Mann–Whitney *U* Test

^a^One month after the cessation of chemotherapy

^b^ *P* value is reported based on the Mann–Whitney *U* Test (for changes of values during the intervention period)

^c^Percent change in proportion to the baseline values

### Proinflammatory cytokines

At the end of supplementation period (one month after the cessation of chemotherapy), the serum levels of IL-6 and TNF-α decreased 20.91 and 12.50 % respectively in the n-3 PUFAs group while they were increased in the placebo group without statistically significant differences. The serum level of hs-CRP was decreased in both groups although it was further reduced in the placebo group (61.02 % vs. 55.21 %, Table 4).

**Table 4:** Serum levels of proinflammatory cytokines in two groups of patients

	n-3 PUFAs (Median, percentile 25–75)	Placebo (Median, percentile 25-75)	*P*-value
IL-6^a^ 1(pg/ml)	28.70 (20.50–42.60)	21.50 (15.20–51.10)	.194*
IL-6 2	22.70 (17.10–30.45)	25.50 (16.80–55.40)	.396**
% Change	−20.91	18.60	
TNF-α^b^ 1(pg/ml)	4.00 (3.60–5.25)	4.30 (3.60–5.60)	.218*
TNF-α 2	3.50 (3.15–4.97)	4.35 (3.15–4.97)	.097**
% Change	−12.50	1.16	
hs-CRP 1(mg/l)	2.95 (.92–7.30)	2.88 (1.31–15.40)	.505*
hs-CRP 2	1.15 (.82–2.32)	1.29 (.82–2.90)	.390**
% Change	−61.02	−55.21	

*P value is reported based on the Mann–Whitney *U* Test

**P value is reported based on the Mann–Whitney *U* Test (for changes of values during the intervention period)

^a^Interleukin-6

^b^Tumor necrosis factor-α

## Discussion

We assessed the efficacy of n-3 PUFAs, mainly DHA, for preventing and reducing the severity of OXIPN in patients with colon cancer. Patients undergoing chemotherapy with oxaliplatin (XELOX regimen) randomly assigned to receive n-3 PUFAs pearls or placebo during the treatment courses and one month after the cessation of therapy (about 25 weeks) while incidence and severity of OXIPN were assessed in both time. This study showed that n3- PUFAs are protective against OXIPN in patients with colon cancer. Here we focused solely on chronic OXIPN that is a sensory- motor axon loss and the major dose-limiting side effect of oxaliplatin. It begins at a cumulative dose of 750–850 mg/m^2^ and may be continued for a long time after the end of treatment [7, 17].

Clinical and electrophysiological finding were summarized by TNSr [15, 18] (ranging 0 to 28). It can be easily used for determining the incidence and severity of CIPN and it has a significant correlation with the known oncologic toxicity scales such as NCI-CTC, Ajani and ECOG scales, and the complete version of TNS as well [14, 15]. It is an effective easy-to-use alternative for TNS.

Nerve conduction studies can assess the extent of chemotherapy-induced peripheral neuropathy objectively and are also capable of identifying patients with subclinical neuropathy before the manifestation of clinically significant peripheral neuropathy [19]. In this study, the a-CMAPs of all three tibial, peroneal and ulnar motor nerves, were increased in proportion to the baseline values in the n-3 PUFAs group, while they decreased in the placebo group (except for peroneal nerve which also increased slightly in the placebo group) without statistically difference between two groups probably due to small sample size (Table 2). Notably, the change percent of a-CMAPs in motor nerves was much less than 50 % indicating that OXIPN is more sensory than motor neuropathy. The a-SAPs of sural and ulnar nerves were decreased in proportion to the baseline values in both the n-3 PUFA and placebo groups. However, the decrease in a-SAP was much greater in the latter group (Table 3). Argyriou et al. reported that the only independent predictor for unfavorable neurological outcomes was the decrease of sural a-SAP >50 % in proportion to the baseline values in cancer patients undergoing treatment with cisplatin and paclitaxel [18]. In this study, DHA helped prevent a sharp reduction in sural (−36.68 % vs. −100.00 %) and ulnar (−18.28 % vs. −42.06 %) a-SAPs in the n-3 PUFAs supplemented group and it improved the a-CMAP of tibial, peroneal and ulnar motor nerves indicating the beneficial effects of n-3 PUFAs (particularly DHA) on both sensory and motor nerve conduction parameters.

Recently, dysfunction of axonal membrane Na^+^ and Ca^2+^channels have been proposed as one of the mechanisms underlying OXIPN as well as pain related to chronic neuropathy [20, 21]. Therefore, finding an agent capable of modulating ion channel activity may be effective in preventing and reducing OXIPN, as some evidences suggested that calcium channel blockers could reduce acute OXIPN in patients receiving modified FOLFOX chemotherapy regimen [22]. Previous studies have shown that the anti-hypertensive as well as antiarrhythmic properties of n-3 PUFAs are due to their ability to block voltage-gated sodium channels (VGSCs) and voltage-gated calcium channels (VGCCs) reversibly in arteries of muscle cells and cardiomyocytes without frequency –dependent resistance [9, 23, 24]. N-3 PUFAs can also block L-type VGCC and VGSCs of the pain neurons involved in neuropathic pain as well [9].

Some studies revealed that an infusion of oxaliplatin in CRC patients caused the release of inflammatory cytokines such as interleukin 6(IL-6) and tumor necrosis factor alpha (TNF-α) [25, 26] Interleukin-1beta (IL-1 β), IL-6 and TNF-α (proinflammatory cytokines) and prostaglandin E2 (PGE2) as an eicosanoid develop neuropathic and inflammatory pain by their direct and indirect impact on the central and peripheral nervous systems (including DRG neurons, and glial cells) [9] . Moreover, IL-1 β and TNF-α contribute to the rapid excitation of nociceptors via the induction of Cyclooxygenase 2 (COX-2) [27]. Serum levels of IL-6 and TNF-alpha were decreased in n-3 PUFAs supplemented group, although there was not a statistical difference with placebo, possibly due to low dose and relatively short period of n-3 PUFAs supplementation.

DHA has anti-neuroinflammatory properties both in central and peripheral nervous system and it has been attributed to the DHA efficacy in the prevention and treatment of neurodegenerative disorders such as major depression, Alzheimer’s and Parkinson’s diseases. Neuroprotectin D1 (NPD1) is synthesized from DHA in the body and it is an effective neuroprotective agent for the prevention and treatment of confirmed neuropathic pain. Noteworthy, the repeated administration of opioids may cause antinociceptive drug tolerance and even hyperalgesia, but DHA-derived NPD1do not create such a resistance even after a long-term use of DHA [28–30]. It can promote axonal regeneration after chemotherapy-induced peripheral neuropathy as a nerve growth factor. NPD1 reduces gene expression of proinflammatory cytokines (IL-1β and TNF-α) and COX-2 as well. Serum levels of IL-6 and TNF-alpha were decreased in n-3 PUFAs supplemented group, although there was not a statistical difference with placebo, possibly due to low dose and relatively short period of n-3 PUFAs supplementation.

In a number of randomized controlled trials using n-3 PUFAs, the rate of adverse events was not greater than 5 % with no considerable difference in frequency with placebo groups and only some GI discomforts like nausea and a fishy burp were reported. The standardized preparation of n-3 PUFAs is in the capsular form and it can be taken without considerable concern for interaction with other medications. We recommended to our patients to take n-3 PUFAs capsules with meals and keep them in the refrigerator. They had a strawberry taste, and were well tolerated by the patients. The US Food and Drug Administration (FDA) recommended maximum levels of 3 g per day of n-3 PUFAs supplementation [31]. In the current study, the total dose of n-3 PUFAs administered was 1244.1 mg per day (640 mg: 54%DHA, 10 % EPA, t.i.d) which was far below the maximum FDA recommended for more prudence. No considerable side effects were reported by the patients.

Among a wide variety of compounds assessed for the prevention or treatment of chemotherapy-induced neuropathy, including vitamin E [32], Ca- Mg infusion [33–35], amifostine, glutamine, and glutathione [36], the Ca- Mg infusion was effective in this field. There is a concern, however of interference with the clinical efficacy of chemotherapy. Other compounds such as antidepressants, anticonvulsants [36] and green tea [37] had limited effects or they were not studied in RCTs.

Lack of long-term follow up of the patients due to high cost needed was one of the limitations of this study. Therefore, coasting phenomenon and self-improvement after the cessation of chemotherapy was not compared between the two study groups. Moreover, placebo did not smell fish oil and it may be considered as another limitation of this study. Despite the relatively small sample size of this study (we could not run a multicenter RCT, because of some problems in coordination between centers), the incidence and severity of OXIPN was different between the n-3 PUFAs and placebo groups, maybe due to the strong effect of DHA on the peripheral nervous system of the patients through the different mechanisms pointed out here and other presently unknown reasons, as the precise mechanism of OXIPN is not clear.

## Conclusions

OXIPN will continue for a long time in patients who received oxaliplatin and it has a remarkable influence on the patients’ quality of life [1, 7]. As the survival of this group of patients is increased significantly worldwide, finding an effective prophylactic or symptomatic therapy for OXIPN should be of special interest [2, 5]. In this study, n-3 PUFAs supplementation could reduce the incidence and severity of OXIPN in patients with colon cancer. It is well tolerated by patients because of few if any complications. Therefore, given the low-cost and safety of DHA, it can be recommended as a prophylactic and therapeutic agent for OXIPN. Further studies with larger sample size and longer follow-up are needed to confirm these findings.

## Abbreviations

a-CMAP, amplitude of compound muscle action potential; a-SAP, sensory action potentials; CIPN, chemotherapy-induced peripheral neuropathy; CRC, colorectal cancer; DHA, docosahexaenoic acid; DML, distal motor latency; EPA, eicosapentaenoic acid; MCV, motor conduction velocity; NCVs, the nerve conduction studies; OXIPN, oxaliplatin-induced peripheral neuropathy; PUFAS, n-3 polyunsaturated fatty acids; TNSr, reduced total neuropathy score
